# Outcomes from a hybrid implementation-effectiveness study of the living well during pregnancy Tele-coaching program for women at high risk of excessive gestational weight gain

**DOI:** 10.1186/s12913-022-08002-5

**Published:** 2022-05-03

**Authors:** Susan de Jersey, Nina Meloncelli, Taylor Guthrie, Hilary Powlesland, Leonie Callaway, Angela T. Chang, Shelley Wilkinson, Tracy Comans, Elizabeth Eakin

**Affiliations:** 1 Department of Nutrition and Dietetics, Royal Brisbane and Women’s Hospital, Metro North Hospital and Health Service, Brisbane, Australia; 2 The University of Queensland, Centre for Clinical Research and Perinatal Research Centre, Faculty of Medicine, Brisbane, Australia; 3 Women’s and Newborn Services, Royal Brisbane and Women’s Hospital, Metro North Hospital and Health Service, Brisbane, Australia; 4 Centre for Allied Health Research, Royal Brisbane and Women’s Hospital, Metro North Hospital and Health Service, Brisbane, Australia; 5 School of Human Movement and Nutrition Sciences, The University of Queensland, Brisbane, Queensland Australia; 6 Mothers, Babies and Women’s Theme, Mater Research Institute - The University of Queensland, Brisbane, Queensland Australia; 7 Centre for Health Services Research, Faculty of Medicine, The University of Queensland, Brisbane, Queensland Australia; 8 School of Public Health, Faculty of Medicine, The University of Queensland, Brisbane, Queensland Australia

**Keywords:** Implementation study, Pregnancy, Gestational weight gain, Nutrition, Telephone counselling, Dietitian

## Abstract

**Background:**

Excess gestational weight gain (GWG) is associated with short-term perinatal complications and longer term cardiometabolic risks for mothers and their babies. Dietitian counselling and weight gain monitoring for women at risk of high pregnancy weight gain is recommended by clinical practice guidelines. However, face-to-face appointments, during a time with high appointment burden, can introduce barriers to engaging with care. Telephone counselling may offer a solution. The Living Well during Pregnancy (LWdP) program is a dietitian-delivered telephone coaching program implemented within routine antenatal care for women at risk of excess GWG. This program evaluation used a hybrid implementation-effectiveness design guided by the RE-AIM framework to report on the primary outcomes (reach, adoption, implementation, maintenance) and secondary outcomes (effectiveness) of the LWdP intervention.

**Methods:**

The LWdP program evaluation compared data from women participating in the LWdP program with a historical comparison group (pregnant women receiving dietetic counselling for GWG in the 12 months prior to the study). The primary outcomes were described for the LWdP program. Between group comparisons were used to determine effectiveness of achieving appropriate GWG and pre and post intervention comparisons of LWdP participants was used to determine changes to dietary intake and physical activity.

**Results:**

The LWdP intervention group (*n* = 142) were compared with women in the historical comparison group (*n* = 49). Women in the LWdP intervention group attended 3.4 (95% CI 2.9–3.8) appointments compared with 1.9 (95% CI, 1.6–2.2) in the historical comparison group. GWG was similar between the two groups, including the proportion of women gaining weight above the Institute of Medicine recommendations (70% vs 73%, *p* = 0.69). Within group comparison showed that total diet quality, intake of fruit and vegetables and weekly physical activity were all significantly improved from baseline to follow-up for the women in LWdP, while consumption of discretionary food and time spent being sedentary decreased (all *p* < 0.05).

**Conclusion:**

The LWdP program resulted in more women accessing care and positive improvements in diet quality, intuitive eating behaviours and physical activity. It was as effective as face-to-face appointments for GWG, though more research is required to identify how to engage women earlier in pregnancy and reduce appointment burden.

**Supplementary Information:**

The online version contains supplementary material available at 10.1186/s12913-022-08002-5.

## Background

Excess gestational weight gain (GWG) is one of the most common adverse outcomes of pregnancy, experienced by half to three quarters of women in developed countries [1, 2]. It is associated with hypertensive disorders of pregnancy, gestational diabetes mellitus (GDM), instrumental and caesarean deliveries, large for gestational age babies and macrosomia [1, 3–5]. Longer term consequences of gaining more weight than recommended during pregnancy include future childhood overweight for offspring and post-partum weight retention leading to obesity in mothers [6, 7]. Each 1 kg of excess GWG has been associated with a 3% increase in childhood obesity [8]. Women with excess GWG retain on average 2 to 4 kg per pregnancy, further contributing to high body weight in women of a reproductive age [6, 7].

Evidence is now well established that theory-based behaviour change nutrition interventions coupled with weight monitoring are effective in reducing GWG [9, 10] and can impact on selected pregnancy outcomes such as GDM and hypertension [11, 12]. In line with this many clinical practice guidelines recommend consideration of [13], or a referral to a dietitian [14] to support women, particularly those with an increased pre-pregnancy body mass index (ppBMI), achieve a healthy pregnancy weight gain. However, uptake to such services in routine care has been poor, particularly with face-to-face care with between 20 and 50% of referred women not attending any appointments [15–18],

Telephone counselling has the potential to provide individualised intensive support to those women at high risk of excess GWG without the need for additional attendance at hospital or antenatal clinics and has been an effective and cost-effective solution for weight management outcomes in many adult populations [19–21]. While telephone based interventions have been trialled in pregnancy [22], there has been limited evaluation of the implementation or effectiveness associated with translation into practice [23].

The Living Well during Pregnancy (LWdP) program was a dietitian delivered telephone coaching program implemented within routine antenatal care. A core program component was motivational interviewing with a foundation in behaviour change principles.

Using the RE-AIM framework [24] to guide evaluation, here we report on the primary outcomes (program reach, adoption, implementation, maintenance) and secondary (effectiveness) outcomes including behavioural and anthropometric changes.

## Methods

### Setting

The study was conducted at a large, tertiary metropolitan hospital that delivers approximately 4500 babies per year. Detailed methods for the LWdP program and evaluation have been previously reported [25].

### Study design

The LWdP program was evaluated using a type II hybrid implementation-effectiveness design [26] which equally emphasises clinical/effectiveness outcomes and implementation outcomes. Data from consenting participants enrolled in the LWdP program were compared with a historical comparison group of pregnant women referred for dietetic support for healthy pregnancy weight management.

### Participants and referral pathways

Participants were clinician or self-referred to the LWdP program for dietetic care between February 2018 and March 2019.

#### Eligibility criteria

Included women were those aged ≥16 years, without pre-existing diabetes and referred for dietetic weight management care during the study period. Specifically, women with a pre-ppBMI greater than 25 kg/m^2^, or a ppBMI less than 25 kg/m^2^ and had been gaining weight above the Institute of Medicine (IOM) [5] recommendations were accepted upon referral at any point in their pregnancy. Only participants who were able to speak and read English sufficiently to allow program participation were included. Those women who did not meet these criteria were provided with appropriate face-to-face dietetic care and did not participate in this evaluation. Women were referred to the program by their antenatal care provider or were able to self-refer into the program through a dedicated program website.

The historical comparison group were pregnant women who met the inclusion criteria, referred for dietetic care in the 12 months prior to the study period.

#### The Living Well during Pregnancy program

A detailed description of the LWdP program is found elsewhere [25]. Briefly, the program was aimed at supporting women at high risk of excess weight gain during pregnancy to achieve GWG within recommendations [5]. The program was grounded in Social Cognitive Theory constructs of self-efficacy, social support and outcome expectancies [27] and emphasised developing skills in behaviour change strategies – goal setting, self-monitoring, identification of potential barriers and problem solving, identifying social support, stimulus control, mindful eating, positive self-talk and self-reward. The goal was for women to track within their healthy GWG for their ppBMI based on IOM recommendations [5] through changing eating and activity behaviours. This included healthy eating, and physical activity, consistent with dietary and physical activity guidelines for pregnancy. Women were eligible for up to 10 telephone coaching calls over their pregnancy and were provided with a participant workbook. Accredited Practising Dietitians with experience in providing antenatal care services who had undergone additional training in motivational interviewing were trained specifically for the delivery of the program. A key component of the program was continuity of care through allocation of the same Dietitian throughout the duration of the program. The program was adapted for pregnancy from the *Healthy Living after Cancer* program [28].

#### Historical comparison group

The historical comparison group were provided with usual dietetic care. Women were offered an individual appointment with the dietitian in a dedicated clinic within the maternity outpatient department. An initial appointment was booked for 40 minutes with review appointments allocated 20 minutes. There was no guarantee of the same dietitian at a review appointment, and these were booked based on clinical judgement in negotiation with the woman. Clinical practice guidelines at the time recommended women had a consultation with a dietitian to support healthy eating in line with the Australian Dietary Guidelines and encourage adherence to weight gain recommendations [14].

### Data collection

Data collection is reported in greater detail elsewhere [25]. The data collection period for the historical group was from October 2016 to October 2017. The data collection for the LWdP group commenced when the program was initiated in February 2018 and the last included participant was enrolled in August 2019. The data for the historical comparison group were extracted from administrative (referral and attendance), medical and pregnancy handheld records (anthropometric and demographic). Data for the LWdP intervention group was collected by study-trained dietitian research assistants using the Research Data Capture (REDCap) online data capture tool to record details of each telephone counselling session including progress, clinical information, and call fidelity (self-reported after each call by clinician via checklist). Behavioural and anthropometric data were collected via an online questionnaire through a health service consumer portal, with the follow-up questionnaire containing additional participant satisfaction and feedback questions. Women who withdrew from the program were sent an online survey to assess opportunities for program improvement and reasons for withdrawal. Administrative data (both routinely collected and purposefully requested for the purposes of the study) were recorded in an excel spreadsheet. An online and paper-based staff survey was sent to all antenatal staff between December 2018 and January 2019. This survey examined staff satisfaction with the LWdP program and referral processes in addition to understanding of eligibility criteria and model of care.

The Fat and Fibre Behaviour Questionnaire (FFBQ) [29], a validated 20 item questionnaire to assess dietary behaviours was used to calculate Fat Index, Fibre Index and Total Diet Index (scored 1–5, with 5 indicating more optimal eating behaviours). The FFBQ was also used to describe the servings of fruits, vegetables, low fat dairy foods and discretionary foods consumed daily. The Intuitive Eating Scale-2 was used to measure participants ability to follow their physical hunger and satiety cues when determining when, what, and how much to eat [30]. The Active Australia Survey [31] was used to assess the frequency and duration of physical activity (walking, moderate and vigorous) in the past week. Total number of sessions and minutes of physical activity were treated as continuous variables. Sedentary behaviour (sitting time per week on weekdays and weekends), questions from the International Physical Activity Questionnaire was used to assess total sedentary time per week [32]. Behavioural outcomes were not available for the historical comparison group as they were not collected as part of routine care.

### Outcomes

Primary outcomes were mapped to the RE-AIM framework measures of *Reach* and *Representativeness*, *Adoption. Implementation,* and *Maintenance.* The RE-AIM measure of *Effectiveness* was a secondary outcome which included anthropometric measures and behaviour change outcomes. Results have been reported in this order.

### Statistical analysis

Analyses were performed using Statistical Package for Social Sciences (Version 20, SPSS Inc., Chicago, USA). All available data were included in analysis. Primary outcomes were reported descriptively. The *adoption* and *reach* primary outcomes were compared to the historical comparison group while the *implementation* outcomes were reported for the LWdP intervention group only. Implementation outcomes also report strategies used according to Expert Recommendations for Implementing Change taxonomy [33]. For the *effectiveness* outcome of GWG, the historical comparison group and LWdP intervention group were compared. GWG differences between the groups was analysed according to ppBMI (continuous and according to meeting the IOM recommendations). Within group GWG differences (LWdP group only) were analysed according to: per protocol and intention-to-treat (where per protocol was defined a priori by the research team as completion of 4 or more telephone counselling sessions which signifies the number of appointments covering the core lifestyle advice and the intensive phase of the program) and whether counselling commenced early in the pregnancy (≤ 16 weeks gestation) vs after 16 weeks gestation. Logistic regression was used to calculate the odds ratio of women in the LWdP intervention group achieving the IOM GWG recommendations and adjusting for pre-specified confounders. Missing GWG data for the LWdP group occurred for less than 15% of participants (*n* = 20). Therefore, mean substitution or data imputation was not performed.

For all other effectiveness outcomes (diet and physical activity behaviours), baseline and follow-up questionnaire data were compared within the LWdP intervention group only and mean change or % change were compared using paired-sample t tests. Comparisons between women completing both baseline and follow-up questionnaires and those who only completed the baseline questionnaire was analysed to understand whether there were differences in characteristics between completers and non-completers. As incomplete behavioural questionnaires at follow-up were greater than completed questionnaires, we chose not to impute data. Only data for those women who completed both questionnaires were include for analysis.

Mean and standard deviation (SD) were reported for normally distributed data while median and interquartile range (IQR) for skewed data. Differences between groups were assessed using independent sample t-tests for continuous variables and chi-squared for categorical variables.

## Results

### Reach and representativeness

**Fig. 1:**
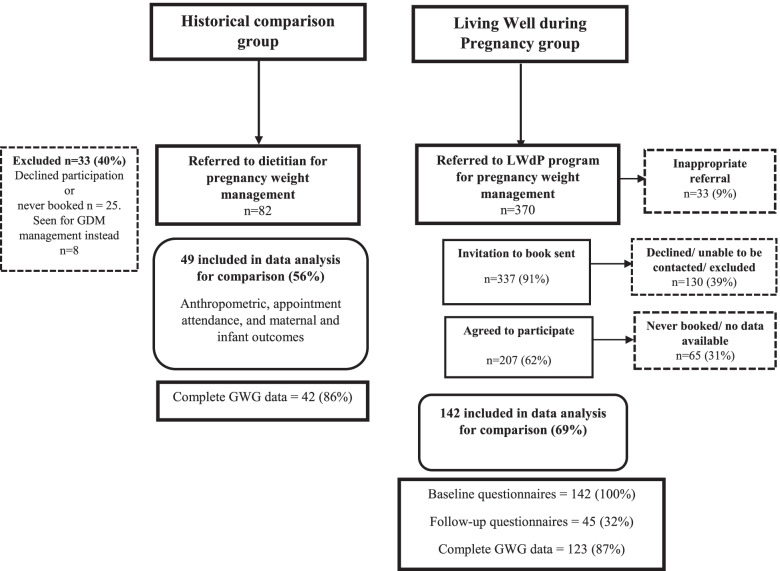
Study flow diagram (reach) of historical comparison and LWdP groups. Percentages relate to the number of participants in the previous level of the flow chart

Referral, participation and exclusion rates for both groups are shown in Fig. 1. During the historical comparison data collection period, 2332 women receiving antenatal care at the study hospital had a ppBMI > 25 kg/m^2^,compared with 2532 women during the LWdP data collection period. Therefore ~ 3.5% of eligible women in the historical comparison group were referred to a dietitian for pregnancy weight management, compared with a ~ 15% during the LWdP study period. Women commenced dietitian appointments at similar weeks gestation across both groups. Participant characteristics of the historical comparison and LWdP intervention groups are shown in Table 1. No meaningful (> 10%) between group baseline differences were apparent.

**Table 1:** Maternal characteristics of participants in the LWdP intervention and historical comparison group

	LWdP intervention group (*n* = 142)	Historical comparison group (*n* = 49)	*P*
Age, years	31.6 (5.2)	29.8 (6.3)	.05
Country of birth (Australia)	88 (62%)	27 (55%)	.40
Nulliparous	81 (57%)	34 (69%)	.13
Education			
Primary/ high school	46 (32%)	–	
Trade certificate or diploma	32 (23%)	–	
University degree (including postgraduate)	64 (45%)	–	
Pre-pregnancy BMI kg/m^2^	30.6 (6.6)	33.4 (7.9)	.02
Underweight (BMI < 18.5 kg/m^2^)	1 (0.7)	0	.56
Normal weight (BMI18.5–24.9 kg/m^2^)	24 (17%)	4 (8%)	.13
Overweight (BMI25–29.9) kg/m^2^)	49 (35%)	14 (29%)	.44
Obese (BMI > 30 kg/m^2^)	68 (48%)	31 (63%)	.07
Gestation at first dietitian appointment, weeks	21.5 (6.2)	22.9 (5.0)	.18

*LWdP* Living Well during Pregnancy, *BMI* Body Mass Index

Results shown as either *n* (%) (categorical) or mean (SD) (continuous)

### Adoption

Seventy-three percent (*n* = 270) of the women referred to the LWdP program were referred by midwives, 23% (n = 86) were self-referrers, 1% (*n* = 3) were referred by General Practitioners, 1% (*n* = 3) by an Obstetrician, < 1% (n = 2) were by a dietitian.

#### Staff survey (referral practices and staff attitudes towards the program)

Fifty-three staff answered the LWdP evaluation survey. Forty-three (77%) respondents were health professionals (42 were midwives/nurses, 2 were Obstetrics and Gynecology doctors) and the remaining 19% (*n* = 10) of respondents were administration officers.

Sixty percent (*n* = 6) of the administration officers and 86% (*n* = 37) of health professional respondents agreed that they understood what the LWdP program was and 100% correctly identified the LWdP description. Education sessions or emails from dietitians, posters/flyers and dietitian referral forms were the most common ways the administration officers learnt about the program and three health professionals said that they learnt about the program from their patients. Fifty percent (*n* = 5) of administration officers agreed that the referral processes were easy. Almost all health professionals agreed that they were aware of who was eligible, how to refer and that referral to the program was easy. More than half of the health professionals (67%) reported they ‘always’ or ‘often’ referred eligible women. Two respondents did not feel comfortable recommending the program. Three administration officers thought they had enough knowledge of the program to answer women’s questions while a further three did not feel they had enough knowledge.

Eighty-three percent (*n* = 44) of survey respondents did not feel as though the burden of appointments or appointment timing would be a barrier to referring to the program. However, more than half (*n* = 24) felt that a barrier to referral (always, often or sometimes as responses) was that women don’t see weight as a priority. Seventeen respondents (32%) thought that women sometimes (or often) had a negative view of dietitians.

### Implementation and maintenance

LWdP delivery required an 80% full time equivalent dietitian coach (senior level). A second coach covered vacation time if leave extended beyond 2 weeks. The coaching calls were offered across 4 days per week with two early morning starts (7.30 am-3.30 pm) and two later evening (9:30 am − 6:00 pm) to enable maximum participation. The time allocation and LWdP program continues to be offered within routine care to all women meeting the referral criteria at the study site. Of note from the staff survey, almost half (*n* = 18) of the respondents did not feel they were informed of the progress of women they referred to the program and the majority would be happy to receive an email or read patient progress notes to keep informed.

#### Completion rates and number of appointments attended

In the historical comparison group, the mean number of appointments was 1.9 (95% CI, 1.6–2.2). The greatest number of appointments attended by a single participant was five. The average number of LWdP appointments attended was 3.4 (95% CI, 2.9–3.8). One participant attended 11 appointments in total. The number of women attending at least four appointments (considered ‘per protocol’) was 58 (41%). There was no ‘per protocol’ attendance difference between women who had self-referred to the LWdP program compared with clinician-referred women (44% vs 40%, *P* = .64, results not shown). Eighty-four women (60%) withdrew from the program before completing at least four appointments. Forty-seven women (34%) attended one or fewer appointments. Women commencing pregnancy overweight had a tendency towards completing four or more appointments compared with women who commenced pregnancy at a normal or obese BMI (47% vs 36% and 38%, respectively, *P* = .55, results not shown).

#### Reasons for withdrawal

Nine women completed the drop-out survey, with the most common reason (*n* = 4) for program withdrawal being ‘*I don’t have enough time to complete calls’*. Competing work priorities, early labour and wanting specific snack/meal suggestions were reasons for not completing the program.

#### Implementation strategies

Twenty-six implementation strategies [33] were used to support service change ranging from accessing new funding to mandating change. A full description of each strategy is outlined in Additional file 1. Six months after the program commenced, a text message to each woman registering to birth at the hospital was sent with information about the program and a link to the self-referral website. In the 3 months before the text message was initiated there was an average of 47 visits to the website per month, increasing to 148 visits per month after the text message (*P* = .03) with self-referrals increasing from 3 per month to 21 per month (*P* < 0.01).

#### Coaching call fidelity

Self-reported coaching call fidelity showed that all topics were covered as planned in the first coaching call for 95% of the participants, however there was no fidelity check undertaken for 15% (*n* = 21) of participants. For each call, goal attainment, goal setting and pre-reading requirements were discussed with more than 80% of participants. Adherence to other planned topics of discussion declined with each subsequent call. For example, the topics discussed with more than 75% of participants in call two were dietary trackers, healthy diet and goal setting whereas reducing fat intake, food safety and healthy portions was discussed less than 50% of the time. For call three, the topics relating to physical activity were discussed with around half of the participants (or fewer) and women appeared to continue the discussions from call two rather than stick to the planned topics. Participants were keen to discuss certain topics earlier than were originally planned in the program’s workbook or felt that certain sections didn’t apply to them (for example, physical activity). By call four, fidelity checks were performed on less than 60% of calls (*n* = 36 out of 60 participants) and only 19% (*n* = 7) of participants discussed planned topics (healthy GWG, meal planning, eating at the right times and tracking weight gain). Generally, call four was focused on topics intended for previous weeks as participants regularly had less time to dedicate to each call than what was originally planned, making it difficult to cover the required number of topics.

#### Participant satisfaction

Thirty-one percent (*n* = 44) of participants completed the satisfaction survey. Respondents were very satisfied or satisfied overall (93%, *n* = 41), with the coaching quality (98%, *n* = 43) and the participant workbook (93%, n = 41). Twenty-four participants (55%) read all the participant workbook while 16 (36%) read most of it.

Comments regarding improvements to the coaching and workbook were positive confirmations of the helpfulness of the program, dietitian counsellors and program flexibility. Participants suggested an electronic workbook (for ease of access when in a telecoaching call), smartphone app or additional videos could enhance the program.

Reported benefits of the program included feeling more positive about mindful eating and keeping on track with healthy eating and exercise. Many women felt they were better able to maintain their weight.

“*I think I have managed to keep my weight within an acceptable amount and have definitely done more exercise than previous pregnancies”*

Very few participants reported any dislikes or improvements for the program. A few participants would have liked additional meal plans while others wished that the program would continue past pregnancy.

*“More resources on meal plan ideas (maybe there was I just didn’t ask)”*

### Effectiveness

In the LWdP intervention group, 100% of women completed the baseline questionnaires. Follow-up behavioural questionnaires were complete for 45 women (32%). There were no differences in age, country of birth, parity or total GWG for women who completed vs non-completers of questionnaires (results not shown). There were significantly more women who commenced pregnancy at an overweight BMI (47% vs 29%) and fewer women in the obese BMI category (36% vs 54%, *P*.046, results not shown) in the group who completed both questionnaires compared to non-completers. Women who had completed the baseline and follow-up behavioural questionnaires attended more dietitian appointments (4.5 ± 3.0 vs 2.8 ± 2.3, *P* = .003, results not shown).

#### Diet quality

Total diet quality (Fat, Fibre and Total Index scores), and fruit and vegetable consumption improved significantly from baseline to follow-up (Table 2). The consumption of discretionary foods (take away, chocolate, chips etc) and sugar-sweetened beverages was reduced at follow up across all categories (Table 2).

**Table 2:** FFBQ Index and dietary intake at baseline and follow up for women participating in the LWdP program

	Baseline	Follow-up	Mean change (95% CI) or % change	*P*
Fat and Fibre Behaviour Index				
Total Index	3.03 (0.45)	3.53 (0.36)	0.51 (0.39, 0.63)	<.001
Fat Index	3.01 (0.59)	3.51 (0.47)	0.50 (0.34, 0.66)	<.001
Fibre Index	2.90 (0.63)	3.43 (0.52)	0.50 (0.37, 0.64)	<.001
Serves of vegetables				<.001
1–4 serves	45 (100%)	28 (62%)		
5 or more serves^a^	0 (0%)	17 (38%)	38%	
Serves of Fruit				.09
0–1 serves	15 (33%)	5 (11%)		
2 serves	19 (42%)	25 (56%)		
3 serves	11 (24%)	15 (33%)		
4 or more serves	1 (2%)	0 (0%)		
Meets recommendations (2+ serves)	31 (69%)	40 (89%)	20%	.02
Dairy				
Usually/always choose low fat milk	14 (47%)*	22 (59%)*	13%	.30
Usually/ always choose low fat cream including ice-cream	15 (33%)	26 (58%)	24%	.02
Usually/ always chooses low fat cheese	13 (29%)	24 (53%)	24%	.02
Discretionary foods (≥ once/ week)				
Takeaway	28 (62%)	10 (22%)	−40%	<.001
Hot chips (French fries)	30 (67%)	13 (29%)	−38%	<.001
Pastries, cakes, sweet biscuits	29 (64%)	19 (42%)	−22%	.04
Chocolates or lollies	34 (76%)	23 (51%)	−24%	.02
Sugar sweetened beverages	22 (49%)	9 (20%)	−29%	<.001
Fruit juice or juice drinks	20 (44%)	12 (27%)	−18%	.08

^a^ missing numbers (15 baseline, 8 follow up)

#### Intuitive eating scale

Scores for the Intuitive Eating Scale-2 [30] improved at follow-up on all measures except *Unconditional permission to eat* (Table 3).

**Table 3:** Intuitive Eating Scale scores with mean change at baseline and follow-up for women participating in the LWdP program

	Baseline	Follow-up	Mean change
Unconditional permission to eat	3.08 (0.42)	3.18 (0.67)	0.10 (−0.13. 0.32)*
Eating for physical rather than emotional reasons	3.22 (0.81)	3.74 (0.88)	0.52 (0.30, 0.75)
Reliance on internal hunger/ satiety cues	3.21 (0.77)	3.53 (0.85)	0.32 (0.12, 0.52)
Body-food choice congruence	3.57 (0.77)	3.90 (0.59)	0.33 (0.15, 0.52)
Total intuitive eating score	2.39 (0.94)	3.53 (0.64)	0.14 (−0.29, 0.57)

Differences between baseline and follow-up for all results *p* < .001 except for * (*p* = .50)

#### Physical activity and sedentary behaviour

The minutes per week of all forms of physical activity increased from baseline to follow-up and the reported sedentary time spent on weekdays and weekends also decreased (Table 4). The number of participants meeting the physical activity guidelines increased at follow-up (62% vs 75%, *P =* .17).

**Table 4:** Physical activity and sedentary time before and after the LWdP program

	Baseline	Follow-up	*P*
Total time (all PA, min/ week, median (IQR)	180 (90–250)	240 (140–310)	.007
Sedentary time weekdays, min/day (IQR)	480 (220–800)	300 (180–600)	.095
Sedentary time weekends, min/day (IQR)	360 (200–480)	240 (120–390)	.02
Physical activity guidelines			.37
Sufficient PA (> 150 min/week)	28 (62%)	34 (75%)	
Insufficient PA (1–149 min/week)	13 (29%)	9 (20%)	
Sedentary (0 min/week)	4 (9%)	2 (4%)	

#### Gestational weight gain

Seventy-three percent (*n* = 30) of women in the historical comparison group and 70% (*n* = 86) in the LWdP intervention group gained more weight than was recommended according to the IOM (*P =* .69, Table 5). More women had met or exceeded the IOM weight recommendations prior to the first dietitian appointment in the historical comparison group (*n* = 23, 47%) than the LWdP intervention group (*n* = 36, 31%) (*P* = .10), though the number for both groups was high. The proportion of women exceeding the weight gain recommendations was similar between groups, regardless of ppBMI (Table 5). Between group comparison of women who attended at least 4 appointments (per protocol) gained average 12.2 kg (6.3) vs 14.8 kg (7.5), *P* = .47 (Additional file 2). However, 10% (*n* = 5) of women achieved ‘per protocol’ in the historical comparison group, compared with 35% (*n* = 50) in the LWdP intervention group (results not shown).

**Table 5:** Gestational weight gain for women in the LWdP intervention group and historical comparison group

	LWdP intervention group (*n* = 142)	Historical comparison group (*n* = 49)	*P*
Exceeded IOM guidelines for GWG^a^	86 (70%)	30 (73%)	.69
Normal weight	16 (67%)	4 (100%)	.33
Overweight	31 (72%)	10 (83%)	.43
Obese	38 (64%)	16 (64%)	.93
Total gestational weight gain until 36 weeks, kgs	14.7 (7.6)	13.5 (8.8)	.41
Normal weight	18.6 (4.6)	26.9 (10.3)	.014
Overweight	15.9 (6.7)	17.1 (9.5)	.64
Obese	12.4 (8.3)	9.7 (4.9)	.07

*IOM* institute of medicine, *GWG* gestational weight gain

^a^Adjusted for 36 weeks gestation

Less women classified as obese before pregnancy, exceeded the IOM recommendations if they commenced the LWdP program before 16 weeks gestation (38% vs 74%, *P* = .008, Additional file 2). Referrer type (clinician vs self-referred) did not appear to influence the amount of GWG, except for women who commenced pregnancy in the normal BMI category where there was a trend towards a smaller proportion of women exceeding IOM GWG recommendations when they self-referred (50% vs 88%, *P* = .09, Additional file 2). Within the LWdP intervention group, the adjusted odds ratio of achieving weight gain according to the IOM guidelines was 0.883 (95% CI, 0.386–2.02) (adjusted for parity, ppBMI, presence of oedema and weeks’ gestation commencing LWdP < 16 weeks vs > 16 weeks).

## Discussion

This study has demonstrated the implementation of the LWdP program increased the referral of women eligible for pregnancy weight management support from one in thirty-three to one in eight, with demonstrated improvements in eating and activity behaviours in those who attended. However, despite a facilitated and deliberate implementation effort to integrate the service into antenatal care, the findings indicate further work is needed to ensure more women are provided with early intervention, particularly women with a high BMI at high risk of adverse pregnancy outcomes.

Despite women not commencing the program until mid-way through their pregnancy, the implementation of LWdP resulted in a 10% increase in eligible women being referred for support during pregnancy and a three-fold increase in women receiving care. However, women’s uptake of the program and retention of women was still poor. A similar evaluation of a telephone coaching lifestyle and weight management service during pregnancy, delivered external to the antenatal care health facility, observed less than one in ten women completing the program [34]. Based on previous research [35] the LWdP program was deliberately integrated into women’s antenatal care where the dietitian health coach could see medical notes from other health care providers between appointments and document in medical records to allow other care providers to monitor progress. This alteration in referral and delivery approach resulted in a fourfold difference in completion compared with an external delivery strategy [34] with four in ten women completing the LWdP program.

While health professionals, predominantly midwives, were the primary source of referrals, a simple implementation strategy of a text message to all women registering for care at the facility resulted in a seven-fold increase in self referrals to the program. A consistent barrier reported by health professionals, particularly midwives is a lack of confidence and shame in discussing weight with women [36, 37], and the negative association women have with being referred to a dietitian [37, 38]. This means many women who may benefit from intervention are never aware services exist to support them. Text message reminders have been commonly used to successfully increase attendance at health care appointments and adherence to behaviour change advice [39]. The LWdP program is one of the first to report the effectiveness of a text message prompt to encourage women to initiate their enrolment in a routine service, thus overcoming a key barrier to accessing support and increasing the reach of the program.

While the LWdP program delivered via telecoaching demonstrated equivalent GWG outcomes as achieved in the face-to-face care model of care, the proportion of women gaining more weight than recommended was high. This excess GWG may have been due to the relatively late recruitment of women to the program. Prior to commencing LWdP, one in three women had met or exceeded their recommended GWG for the entire pregnancy. First trimester GWG is recommended at around 1–2 kg regardless of ppBMI, with excess in early pregnancy being most closely associated with adverse outcomes including GDM [40], pre-eclampsia [41] and high birthweight [42]. Interestingly, women who commenced LWdP prior to 16-weeks gestation experienced lower GWG and a lower proportion of women who had a ppBMI in the obese range exceeded GWG recommendations. Furthermore, no additional benefits were noted towards achieving appropriate GWG for women attending per-protocol (4 or more appointments). These findings emphasise the importance of engaging women early in their pregnancy to support appropriate GWG, but to achieve this, barriers must be overcome. ‘Late’ entry to birthing facilities (usually ~ 16 to 18 weeks) often results in a challenging situation of addressing this early excess GWG through lifestyle intervention when the rate of recommended GWG is greater [5]. Potential strategies to address these issues include aligning services with primary care settings to support early pregnancy lifestyle support [43] and providing tailored, individualised schedules of care to appropriately meet women’s needs [44].

The improvements in dietary intake observed in women who participated in the LWdP program are consistent with previous interventions to support health behaviour change in pregnancy that impact on GWG [45, 46]. Significant improvements in dietary quality were observed, driven by a reduction in discretionary food and an increase in fruit and vegetables. While there was no observed effect on GWG, high diet quality in pregnancy has been associated with a reduction in GDM, hypertension and pre-term birth [47]. Furthermore, if sustained, these improvements may contribute to a lowering of long-term diabetes and cardiovascular disease risk in women [48]. Somewhat unique to the LWdP was a focus on intuitive eating. The process of *how* women eat is likely to be as important, if not more so than, *what* women eat if long term behaviour change is sustained. Intuitive eating is considered an adaptive form of eating where there is a connection with internal hunger and satiety cues rather than emotions or cognitions driving food consumption [30, 49]. Developing a healthy relationship with food where there is not a pre-occupation with dieting or the labelling of food as good or bad is needed before healthy eating can be pursued [49]. For many women experiencing a high body weight, breaking a long-held dieting cycle is likely to be important to sustaining behaviour change consistent with healthy eating and weight management. The improvements in intuitive eating observed within the program if sustained may assist women’s eating behaviours well beyond the current pregnancy, having a long- term positive influence. This requires further investigation.

Positive changes in physical activity based on interventions during pregnancy have been mixed and vary according to the provision of supervised and structured activity [50]. The reduction in sedentary time and increase in overall duration of physical activity observed with the LWdP program based on counselling and behaviour change techniques, demonstrates that improved behaviour can be achieved independent of women needing to be provided with additional classes through birthing facilities.

There is limited guidance on the optimal duration, intensity, delivery method for the interventions to support behaviour change and healthy weight gain [10]. This may be because of the complex and individualised nature of lifestyle behaviour change and GWG. The LWdP program was specifically designed with continuity of health care professional to facilitate rapport and person-centred care, with call fidelity reducing as the call number progressed. Comments indicated women wanted to address topics relevant to them earlier than scheduled. Furthermore, a key factor in women withdrawing or not taking up LWdP was time and appointment burden. This is a common reason for not adopting weight management interventions in pregnancy, [34] and while remote delivery removes travel time, it does not address the need for another appointment. Collectively, the lack of evidence for what constitutes optimal care, and in practice the limited ability to meet women’s needs by imposing a rigid schedule, point to the need for flexibility in services and modalities to deliver person centred care. Offering a suite of evidence-based options for remote delivery behaviour change support that can be individualised to each woman’s circumstance may provide solutions to engagement challenges, particularly to those women with a high body weight that may have more comorbidities requiring high risk pregnancy care.

### Strengths and limitations

The findings of this study need to be considered in the context of several strengths and limitations. The completion of behavioural questionnaires by women was low and limited to those more likely to complete the program. The behavioural improvements observed are likely to reflect those women most motivated for change, it is possible that different approaches may be needed for women experiencing greater barriers to change or who are less motivated. Furthermore, the detailed program workbook and telephone delivery may have been a deterrent for women with a lower level of literacy. Future work needs to explore support options for those with lower educational attainment. The historical comparison group received face to face dietetic intervention, limiting the ability to determine the effectiveness of the LWdP program compared to no intervention. It is likely the differences observed in this evaluation would be more profound if compared to no intervention. However, in the context of overwhelming evidence and clinical guidelines recommending dietetic care for weight management support for women, this team deemed it unethical to withhold appropriate treatment. The dissemination methods of the staff survey meant we were unable to identify the total number approached to determine completion rate. A strength of this study was the strong theoretically driven approach, and the applied implementation within routine care demonstrated that a change in model of care is feasible within a large health service and results in favourable behaviour change for women who engage, with high participant satisfaction.

## Conclusions

This is one of the few studies to report on the implementation of a telephone delivered behaviour change lifestyle program incorporated in routine antenatal care. It was developed to translate evidence from successful interventions into practice with facilitated implementation efforts specifically focussed on overcoming barriers to uptake and engagement. The intervention resulted in more women receiving care. Those women who engaged improved their eating and activity behaviours and intuitive eating and was no different than face to face care for GWG outcomes. The program requires adaptation to offer program enhancements such as technology that provide behaviour change support while reducing the appointment burden on women, while retaining the continuity of dietetic carer and the integration into antenatal care. For future scale up, a deeper understanding of how to engage those women with a ppBMI of 30 kg/m^2^ and over earlier in pregnancy is needed to guide this implementation. Broader public health efforts are needed to focus efforts to reduce the prevalence of excess first trimester GWG for all women.

## Supplementary Information

**Additional file 1.** Expert Recommendations for Implementing Change (ERIC) strategies [33] used to facilitate implementation of Living Well during Pregnancy.

**Additional file 2.** Gestational weight gain (exceeding the Institute of Medicine recommendations and total GWG) for women participating in the LWdP program, analysed by appointments (less than four and four or more appointments) and gestational at commencement of program (before or on 16 weeks vs after 16 weeks).

## Data Availability

The datasets analysed for the current study are available from the corresponding author on reasonable request and in accordance to local restrictions governing the privacy of information obtained from medical records.

## Abbreviations

LWdP: Living Well during Pregnancy
ppBMI: Pre-pregnancy body mass index
GWG: Gestational weight gain
IOM: Institute of Medicine
GDM: Gestational diabetes mellitus
FFBQ: Fat and Fibre Behavioural Questionnaire
