# Supplementary material for: Outcomes from a hybrid implementation-effectiveness study of the living well during pregnancy Tele-coaching program for women at high risk of excessive gestational weight gain
Source: BMC Health Serv Res. 2022 May 3;22:589. doi: 10.1186/s12913-022-08002-5 (PMC9063237; doi:10.1186/s12913-022-08002-5)
Supplement: Supplementary file 1 — Additional file 1. Expert Recommendations for Implementing Change (ERIC) strategies [33] used to facilitate implementation of Living Well during Pregnancy. [file 12913_2022_8002_MOESM1_ESM.docx]

**Additional File 1**

| Strategy | Definition^(1)^ | Description of activities |
| --- | --- | --- |
| 1. Access new funding | Access new or existing money to facilitate the implementation | A service development request was submitted and approved for recurrent additional dietitian and administration time to support the program.  Small research grant funding supported evaluation for 12 months. |
| 1. Assess for readiness and identify barriers and facilitators | Assess various aspects of an organization to determine its degree of readiness to implement, barriers that may impede implementation, and strengths that can be used in the implementation effort | 10 years of local research examining care practices in relation to supporting healthy weight gain from consumer and clinician perspectives. Barriers to delivery of care assessed, highlighting lack of funding and administrative support. Consumer’s appointment burden, parking barriers established. Clinical and academic leadership and local context in support of intervention supported service development/business case for implementation. |
| 1. Build a coalition | Recruit and cultivate relationships with partners in the implementation effort | Multidisciplinary workshop engagement  Multidisciplinary working group  Engaged clinicians within models of care internally and GP primary health network liaison |
| 1. Change physical structure and equipment | Evaluate current configurations to adapt, as needed the physical structure and/or equipment (e.g. changing the layout of a room, adding equipment to best accommodate the targeted intervention. | Dual computer screening acquired to facilitate viewing of past medical notes while simultaneously documenting in electronic chart. Headset acquired to allow handsfree consultation. |
| 1. Change record systems | Change record systems to allow better assessment of implementation or clinical outcomes. | Consumer electronic data collection implemented. Electronic capture of clinical notes established. |
| 1. Change service sites | Change the location of clinical service sites to increase access | Service delivery mode changed to telephone |
| 1. Conduct educational meetings | Hold meetings targeted toward different stakeholder groups (*e.g.*, providers, administrators, other organizational stakeholders, and community, patient/consumer, and family stakeholders) to teach them about the clinical innovation | Separate meetings conducted with administration team, midwifery teams and a steering committee of key clinical leaders including GP’s to discuss implementation and promote the program. |
| 1. Conduct local needs assessment | Collect and analyze data related to the need for the innovation | New Beginnings Healthy Mothers and Babies Study undertaken |
| 1. Conduct ongoing training | Plan for and conduct training in the clinical innovation in an ongoing way | Facilitator manual established with training checklist and competencies to be completed prior to independently delivering the program. New team member trained approx. every 6 months. |
| 1. Develop academic partnerships | Partner with a university or academic unit for the purposes of shared training and bringing research skills to an implementation project | Partnered with academic institution with international track record in implementation of telephone delivered interventions for weight management |
| 1. Develop education materials | Develop and format manuals, toolkits, and other supporting materials in ways that make it easier for stakeholders to learn about the innovation and for clinicians to learn how to deliver the clinical innovation | Program workbook developed. Facilitator manual developed. Program website developed with link provided in all promotional and educational materials. |
| 1. Distribute educational materials | Distribute educational materials (including guidelines, manuals, and toolkits) in person, by mail, and/or electronically | Program workbook sent in hardcopy and electronically to consumers prior to first call. Program materials disseminated to GP’s and work areas within the hospital in hardcopy and electronically. |
| 1. Facilitation | A process of interactive problem solving and support that occurs in a context of a recognized need for improvement and a supportive interpersonal relationship | Program manager role established to facilitate program implementation and process changes. Facilitator supported by project team at weekly/fortnightly meetings. |
| 1. Identify and prepare champions | Identify and prepare individuals who dedicate themselves to supporting, marketing and driving through an implementation, overcoming indifference or resistance that the intervention may provoke in an organisation | All maternity dietitians educated on the program to become champions. Each dietitian allocated to an antenatal care team/midwifery model of care and point of contact and champion for the program. Maternity senior dietitian and clinical lead champions for the program internally and externally. |
| 1. Increase demand | Attempt to influence the market for the clinical innovation to increase competition intensity and to increase the maturity of the market for the clinical innovation. | Provided incentives to top refers to the program each month to promote competition within clinicians to refer. Promote program to GP’s within news letters and established communication. Promote self-referral to women through direct text message and social media posts. |
| 1. Inform local opinion leaders | Inform providers identified by colleagues as opinion leaders or “educationally influential” about the clinical innovation in the hopes that they will influence colleagues to adopt it | Clinical leaders formed steering committee for implementation. Working group to support healthy weight development |
| 1. Intervene with patients/consumers to enhance uptake and adherence | Develop strategies with patients to encourage and problem solve around adherence | Direct text messaging to consumers eligible with link to self-referral website established as routine care. Consumers |
| 1. Involve patients/consumers and family members | Engage or include patients/consumers and families in the implementation effort | Consumers engaged to determine optimal timing of service delivery. Satisfaction and feedback from participants requested. |
| 1. Mandate change | Have leadership declare the priority of the innovation and their determination to have it implemented | Service leaders provided additional funding to allow implementation and supported implementation efforts |
| 1. Obtain and use patients/consumers and family feedback | Develop strategies to increase patient/consumer and family feedback on the implementation effort | Consumers who do not complete the program as intended invited to complete online survey. Participants invited to complete satisfaction survey. |
| 1. Organise clinician implementation team meetings | Develop and support teams of clinicians who are implementing the innovation and give them protected time to reflect on the implementation effort, share lessons learned and support one another’s learnings | Weekly implementation team meetings established at beginning of implementation efforts, reduced to fortnightly as progressed. Discussed challenges and progress, opportunities for change to support implementation. Time within project management role provided. |
| 1. Prepare patients/consumers to be active participants | Prepare patients/consumers to be active in their care, to ask questions, and specifically inquire about care guidelines, the evidence behind clinical decisions, or about available evidence supported treatments | Consumers provided with information regarding program prior to first call, including goal setting and expectations for monitoring behaviour. Information about rationale for program provided in workbook and to be discussed by health coach at first call. |
| 1. Purposely re-examine the implementation | Monitor progress and adjust clinical practices and implementation strategies to continuously improve the quality of care. | Regular meeting of local implementation team to discuss progress, review practices and referral numbers. Adjustments made in response to feedback |
| 1. Recruit, designate and train for leadership | Recruit, designate and train leaders for the change effort | Succession planning established with program training checklist and competencies developed. Transition to business as usual with program leadership incorporated into position description of Maternity Senior Dietitian |
| 1. Remind clinicians | Develop reminder systems designed to help clinicians to recall information and/or prompt them to use the clinical innovation | In-services with each midwifery model of care established; program included in GP Alignment program annually; program referral included on hospital “booking in” documentation checklist. |
| 1. Revise professional roles | Shift and revise roles among professionals who provide care, and redesign job characteristics | Shift modification to allow early start and late finishes to facilitate the delivery of care outside usual business hours. |

1. Powell BJ, Waltz TJ, Chinman MJ *et al.* (2015) A refined compilation of implementation strategies: results from the Expert Recommendations for Implementing Change (ERIC) project. *Implementation Science* **10**, 1-14.
