# Supplementary material for: Outcomes from a hybrid implementation-effectiveness study of the living well during pregnancy Tele-coaching program for women at high risk of excessive gestational weight gain
Source: BMC Health Serv Res. 2022 May 3;22:589. doi: 10.1186/s12913-022-08002-5 (PMC9063237; doi:10.1186/s12913-022-08002-5)
Supplement: Supplementary file 2 — Additional file 2. Gestational weight gain (exceeding the Institute of Medicine recommendations and total GWG) for women participating in the LWdP program, analysed by appointments (less than four and four or more appointments) and gestational at commencement of program (before or on 16 weeks vs after 16 weeks). [file 12913_2022_8002_MOESM2_ESM.docx]

**Additional File 2**

Gestational weight gain (exceeding the Institute of Medicine recommendations and total GWG) for women participating in the LWdP program, analysed by appointments (less than four and four or more appointments) and gestational at commencement of program (before or on 16 weeks vs after 16 weeks).

|  | Less than 4 appointments  (n=74) | 4 or more appointments  (n=49) | *P* |
| --- | --- | --- | --- |
| GWG exceeding IOM recommendations | 48 (65%) | 38 (78%) | 0.677 |
| Normal weight | 10 (77%) | 7 (88%) | 0.549 |
| Overweight | 14 (61%) | 17 (85%) | 0.078 |
| Obese | 24 (63%) | 14 (67%) | 0.788 |
| Total GWG, kg, mean (SD) | 14.3 (8.4) | 15.3 (6.0) | 0.448 |
| Normal weight | 18.8 (5.6) | 18.2 (2.7) | 0.803 |
| Overweight | 14.7 (7.7) | 17.4 (5.2) | 0.190 |
| Obese | 12.4 (9.3) | 12.3 (6.5) | 0.947 |
|  | **Commenced  ≤16 weeks (n=29)** | **Commenced >16 weeks**  **(n=94)** |  |
| Exceeding IOM guidelines | 17 (68%) | 69 (73%) | 0.129 |
| Normal weight | 2/2 (100%) | 15/19 (79%) | 0.471 |
| Overweight | 9/11 (82%) | 22/32 (69%) | 0.405 |
| Obese | 6/16 (38%) | 32/43 (74%) | 0.008 |
| Total GWG | 12.9 (7.3) | 15.2 (7.6) | 0.162 |
| Normal weight | 20.5 (3.5) | 18.4 (4.7) | 0.553 |
| Overweight | 15.4 (5.6) | 16.1 (7.1) | 0.758 |
| Obese | 10.1 (7.6) | 13.2 (8.5) | 0.229 |
|  | **Clinician referred (n=93)** | **Self-referred**  **(n=28)** |  |
| Exceeding IOM guidelines | 66 (71%) | 19 (68%) | 0.752 |
| Normal weight | 14/16 (88%) | 2/4 (50%) | 0.094 |
| Overweight | 21/31 (68%) | 10/12 (83%) | 0.307 |
| Obese | 31/46 (67%) | 7/12 (58%) | 0.557 |
| Total GWG | 15.1 (7.9) | 14.3 (5.8) | 0.636 |
| Normal weight | 18.5 (4.7) | 15.3 (3.2) | 0.108 |
| Overweight | 15.8 (7.1) | 16.2 (5.7) | 0.868 |
| Obese | 12.8 (8.7) | 12.0 (6.0) | 0.741 |

IOM Institute of Medicine; GWG gestational weight gain

Referral data missing
